# Determinants of survival and retrospective comparisons of 183 clinical trial patients with myelofibrosis treated with momelotinib, ruxolitinib, fedratinib or BMS- 911543 JAK2 inhibitor

**DOI:** 10.1038/s41408-022-00780-9

**Published:** 2023-01-04

**Authors:** Naseema Gangat, Kebede H. Begna, Aref Al-Kali, William Hogan, Mark Litzow, Animesh Pardanani, Ayalew Tefferi

**Affiliations:** Division of Hematology, Mayo Clinic, Rochester, MN USA

**Keywords:** Myeloproliferative disease, Myeloproliferative disease

## Abstract

Between October 2007 and July 2013, 183 Mayo Clinic patients (median age 65 years; 58% males) with high/intermediate risk myelofibrosis (MF) were enrolled in consecutive phase 1/2 JAK2 inhibitor (JAKi) clinical trials with momelotinib (*n* = 79), ruxolitinib (*n* = 50), fedratinib (*n* = 23) and BMS-911543 (*n* = 31). Using conventional criteria, the respective response rates for spleen and “transfusion-dependent anemia” were 47%, 32%, 83%, 62% and 51%, 30%, 10%, 44%, respectively, favoring momelotinib for anemia response (*p* = 0.02) and fedratinib for spleen response (*p* < 0.01). All study patients were followed to death or 2022, during which time 177 (97%) drug discontinuations, 27 (15%) leukemic transformations, and 22 (12%) allogeneic stem cell transplants (ASCT) were recorded. 5/10-year survival rate for all 183 patients was 41%/16% and not significantly different across the four drug cohorts (*p* = 0.33). Multivariable analysis of pre-treatment variables identified age >65 years (HR 3.5), absence of type 1/like *CALR* mutation (HR 2.8), baseline transfusion need (HR 2.1), and presence of *ASXL1*/*SRSF2* mutation (HR 1.6) as risk factors for overall survival; subsequent HR-based modeling segregated three risk categories with 5/10-year survival rates of 84%/60%, 44%/14%, and 21%/5% (*p* < 0.01). In addition, spleen (*p* < 0.01) and anemia (*p* = 0.01) responses were independently associated with improved short-term survival while long-term survival was secured only by ASCT (5/10-year survival rate 91%/45% vs 47%/19% in non-transplanted patients; *p* < 0.01). The current retrospective study suggests the value of specific pre-treatment variables in identifying long-lived MF patients receiving JAKi and also confirms recent observations on the favorable impact of treatment response on short-term and of ASCT on long-term survival.

## Introduction

Currently, ruxolitinib, fedratinib and pacritinib are three FDA-approved JAK2 inhibitors (JAKi) for treatment of myelofibrosis (MF) [1]. In addition, FDA-approval for momelotinib is awaited. All JAKi provide palliation through similar reduction of splenomegaly and relief of constitutional symptoms but differ in their ability to ameliorate anemia. In a phase-1/2 study of ruxolitinib in MF, ruxolitinib effectively reduced spleen size in 52% of patients [2]. The corresponding spleen response rates in the phase-3 COMFORT-1 (ruxolitinib *vs* placebo) [3], and COMFORT-2 (ruxolitinib *vs* best available therapy) [4], studies were 41.9 and 28%, while symptom response was achieved in 45.9% of ruxolitinib treated patients in the COMFORT-1 study [3]. However, in both studies, patients on ruxolitinib frequently developed anemia (45.2%/42%) [3, 4]. On the other hand, in a phase-1/2 study of momelotinib in MF, momelotinib therapy produced anemia response (54%) and reduction in splenomegaly (40%) [5, 6]. These observations were confirmed in the phase-3 SIMPLIFY-1 [7] (momelotinib *vs* ruxolitinib) and SIMPLIFY-2 studies [8] (momelotinib *vs* best available therapy) with week 24 transfusion independent rates of 66.5 and 43% in the momelotinib arm, respectively. Moreover, spleen response was comparable with momelotinib and ruxolitinib therapy (26.5% vs 29%) [7]. Similar spleen and symptom responses were also observed in phase-1/2 studies of fedratinib (39%/>50%) [9], and BMS-911543 (73%>50%) [10], in MF. These findings were confirmed in the phase-3 JAKARTA-1 study of fedratinib, with spleen and symptom response rates of 36 and 34%, respectively, however, 43% of fedratinib treated patients experienced grade 3 or 4 anemia [11].

The favorable impact of JAKi on splenomegaly and constitutional symptoms, and of momelotinib on anemia in patients with MF is well-established, but whether achievement of response following JAKi therapy positively influences long-term survival is unclear. Accordingly, in the current study, which includes MF patients treated on JAKi clinical trials at the Mayo Clinic, our primary objective was to i) retrospectively compare long-term treatment outcomes in momelotinib, ruxolitinib, fedratinib, and BMS-911543 treated patients with MF, ii) identify clinical and molecular predictors of response, overall and leukemia-free survival, and iii) examine the impact of treatment response on survival.

## Methods

The current study includes JAKi naïve patients with primary myelofibrosis (PMF), post-polycythemia vera and post-essential thrombocythemia MF enrolled in consecutive phase-1/2 momelotinib (NCT00935987), ruxolitinib (NCT00509899), fedratinib (NCT00631462, NCT01420770), and BMS-911543 (NCT01236352) clinical trials between October 2007 and July 2013. The latter clinical trial was terminated by the sponsor in November 2015. Study patients were retrospectively recruited after institutional review board approval with follow-up updated in July 2022. Patient eligibility, study design, drug doses and schedule have previously been published [2, 5, 9, 10]. Diagnosis of PMF, post-ET and post-PV MF were according to conventional criteria [12]. Baseline transfusion status, spleen and anemia response were assessed according to the modified revised international working group for myelofibrosis research and treatment (IWG-MRT) criteria [13]. Screening for *JAK2*, *MPL*, *CALR*, *ASXL1*, *SRSF2, IDH1/2*, and *U2AF1* mutations and dynamic international scoring system (DIPSS)-plus risk stratification and unfavorable karyotype categorization were as previously described [14, 15]. All statistical analyses considered clinical and laboratory variables obtained at the time of study initiation. Conventional statistical methods were applied for response and survival analysis. Survival was calculated from the time of study drug initiation to last follow-up or death and survival curves prepared by the Kaplan-Meier method and compared by the log-rank test. Cox proportional hazard model was used for multivariable analysis. JMP Pro 16.0.0 software package, SAS Institute, Cary, NC was used for statistical analysis.

## Results

### Patient characteristics

A total of 183 JAKi naïve patients with MF (median age 65 years, range 34–89; 58% males, 60% PMF) received momelotinib (*n* = 79, 43%, median dose, 300 mg daily), ruxolitinib (*n* = 50, 27%, median dose, 22.5 mg twice daily), fedratinib (*n* = 23, 13%, median dose, 680 mg daily), and BMS-911543 (*n* = 31, 17%, median dose, 320 mg daily) at a median of 27 months following diagnosis. Driver mutation profile included *JAK2* in 80%, *CALR* in 11% (type 1/like *CALR* in 9%), *MPL* in 5%, triple negative in 4%; other mutations included *ASXL1* in 58/124 (47%), *SRSF2* in 16/84 (19%), *U2AF1* in 3/47 (6%) and *IDH1/2* in 2/74 (3%) of evaluable patients. Karyotype was abnormal in 97 (53%); among the latter, 53% were unfavorable. DIPSS-plus risk distribution was intermediate-1 (15%), intermediate-2 (46%), and high (39%). At the time of study drug initiation, 166 (91%), 66 (36%), and 136 (74%) of patients demonstrated palpable splenomegaly, transfusion-dependent anemia, and constitutional symptoms, respectively. Table 1 provides a comparison of clinical and laboratory characteristics at the time of treatment initiation and response outcomes for MF patients receiving JAKi in the context of a clinical trial. Patients on the momelotinib arm were more likely to be older (*p* < 0.01), transfusion-dependent (*p* = 0.01), harbor type 1/like *CALR* mutations (*p* = 0.09) and belong to DIPSS-plus high-risk category (*p* < 0.01), and less likely to be affected by constitutional symptoms (*p* < 0.01), compared to patients not receiving momelotinib. On the other hand, patients receiving ruxolitinib were more likely to be younger (*p* = 0.02), males (*p* = 0.01), *JAK2* mutated (*p* = 0.04), belong to DIPSS-plus intermediate risk group (*p* < 0.01), and less likely to present with transfusion-dependent anemia (*p* < 0.01). Fedratinib treated patients were more likely to present with leukocytosis (*p* = 0.02) and thrombocytosis (*p* = 0.02) while patients receiving BMS-911543 were more likely to be females (*p* = 0.04) and affected by constitutional symptoms (*p* < 0.01). However, *ASXL1/SRSF2* mutation distribution and median time from diagnosis to initiation of therapy was similar for each treatment group (*p* > 0.1).

**Table 1:** Comparison of clinical characteristics at time of treatment initiation and outcomes for 183 JAK2 inhibitor-naive patients with myelofibrosis receiving *JAK2* inhibitors in the context of clinical trials.

Variables	All patients *N* = 183	Ruxolitinib *N* = 50 (27%) (*P* value vs others)	Fedratinib *N* = 23 (13%) (*P* value vs others)	Momelotinib *N* = 79 (43%) (*P* value vs others)	BMS-911543 JAK2 inhibitor *N* = 31(17%) (*P* value vs others)
Age in years, median (range)	65 (34–89)	62 (39–78) **(0.02)**	64 (47–83) (0.93)	67 (34–89) **(<0.01)**	64 (34–78) (0.21)
Male, *n* (%)	106 (58)	37 (74) **(0.01)**	13 (57) (0.88)	43 (54) (0.40)	13 (42) **(0.04)**
MF type, *n* (%)					
- Post-ET MF	27 (15)	7 (14)	3 (13)	12 (15)	5 (16)
- Post-PV MF	47 (26)	17 (34)	6 (26)	16 (20)	8 (26)
- PMF	109 (60)	26 (52) (0.29)	14 (61) (0.97)	51 (65) (0.33)	18 (58) (0.97)
Driver mutation, *n* (%)	*n* = 175	*n* = 46	*n* = 23	*n* = 79	*n* = 27
- JAK2	140 (80)	42 (84)	21 (91)	56 (71)	21 (78)
*-* CALR	20 (11)	1 (2)	2 (9)	13 (16)	4 (15)
*-* MPL	8 (5)	1 (2)	0	6 (8)	1 (4)
*-* Triple negative	7 (4)	2 (4)	0	4 (5)	1 (4)
Type 1/like CALR vs others		**(0.04)**	(0.86)	(0.09)	(0.79)
Mutations, *n* (%)					
*-* ASXL1	58/124 (47)	16/30 (53) (0.41)	6/10 (60) (0.38)	30/72 (42) (0.18)	6/12 (50) (0.81)
*-* IDH1/2	2/74 (3)	0/25 (0) (0.20)	0/7 (0) (0.53)	2/38 (5) (0.10)	0/4 (0) (0.63)
*-* SRSF2	16/84 (19)	2/15 (13) (0.52)	1/6 (17) (0.88)	13/59 (22) (0.27)	0/4 (0) (0.19)
*-* U2AF1	3/47 (6)	0/7 (0) (0.32)	2/4 (50) **(0.01)**	1/36 (3) (0.10)	0 -
Splenomegaly, *n* (%)	166 (91)	47/49 (96) (0.71)	23 (100) (0.11)	72/77 (94) (0.45)	27/29 (93) (0.64)
Transfusion-dependent, *n* (%)	66 (36)	10 (20) **(<0.01)**	10 (43) (0.43)	37 (47) **(0.01)**	9 (29) (0.37)
Constitutional symptoms, *n* (%)	136 (74)	42 (84) (0.06)	17 (74) (0.96)	46 (58) **(<0.01)**	31 (100) **(<0.01)**
Prior treatment, *n* (%)	144 (79)	45 (90) **(0.02)**	19 (83) (0.62)	57 (72) (0.06)	23 (74) (0.51)
- Hydroxyurea	101 (55)	34 (68)	14 (61)	37 (47)	16 (52)
- ESA	36 (20)	14 (28)	5 (22)	17 (22)	0
- Interferon	15 (8)	9 (18)	1 (4)	4 (5)	1 (3)
- Thalidomide	27 (15)	14 (28)	4 (17)	7 (9)	2 (6)
- Lenalidomide	17 (9)	10 (20)	1 (4)	4 (5)	2 (6)
- Pomalidomide	24 (13)	5 (10)	1(4)	15 (19)	3 (10)
Diagnosis to start of therapy, months, median (range)	27 (0.1–419)	33 (0.2–419) (0.39)	16 (1.4–280) (0.15)	25 (0.1–275) (0.65)	40 (0.3–188) (0.50)
Hemoglobin, g/dl, median (range)	9.9 (6.4–15.3)	10.6 (7.6–15.3) **(0.01)**	9.5 (7.9–14.7) (0.54)	9.6 (6.7–14) **(<0.01)**	9.9 (6.4–14.6) (0.95)
Leukocyte count × 10^9^/L, median (range)	13.7 (1.5–232)	15.6 (2.2–136) (0.83)	25.5 (4–219) **(0.02)**	10.9 (1.5–232) (0.07)	12 (3.2–129.5) (0.63)
Platelet count × 10^9^/L, median (range)	204 (51–1061)	222 (75–774) (0.37)	236 (82–1061) **(0.02)**	169 (51–738) (0.07)	204 (56–636) (0.32)
Circulating blasts %, median (range)	1(0–14)	1 (0–11) (0.48)	2 (0–9) (0.30)	1 (0–14) (0.80)	1 (0–5) (0.10)
Karyotype, *n (*%)					
- Abnormal karyotype	97 (53)	28 (54) (0.62)	11 (48) (0.59)	41 (52) (0.79)	17 (55) (0.82)
- Unfavorable karyotype	51 (28)	13 (26 (0.59)	8 (35) (0.52)	23 (29) (0.97)	9 (29) (0.99)
DIPSS plus risk, *n (*%)					
- Intermediate-1	28 (15)	17 (34)	3 (13)	1 (1)	7(23)
- Intermediate-2	84 (46)	27 (54)	14 (61)	29 (37)	14 (45)
- High	71 (39)	6 (12) **(<0.01)**	6 (26) (0.29)	49 (62) **(<0.01)**	10 (32) (0.45)
Spleen response, *n* (%)	83/166 (50)	15/47 (32) **(<0.01)**	19/23 (83) **(<0.01)**	33/70 (47) (0.53)	16/26 (62) (0.19)
Anemia response in transfusion-dependent patients, *n* (%)	27/66 (41)	3/10 (30) (0.44)	1/10 (10) **(0.02)**	19/37 (51) **(0.04)**	4/9 (44) (0.82)
Symptom response, *n* (%)	8/136 (60)	24/42 (57) (0.54)	11/17 (65) (0.74)	22/46 (48) **(<0.01)**	26/31 (84) **(<0.01)**
Hematological toxicity, *n* (%)	138 (75)	38 (76) (0.91)	20 (87) (0.14)	58 (73) (0.59)	22 (71) (0.53)
Non-Hematological toxicity, *n* (%)	152 (83)	38 (76) (0.13)	21 (91) (0.23)	75 (95) **(<0.01)**	18 (58) **(<0.01)**
Discontinuation of *JAK2* inhibitor, *n* (%)	177 (97)	50 (100)	23 (100)	74 (94)	31 (100)
Time on *JAK2* inhibitor in months (median, range)	*n* = 177	*n* = 50	*n* = 23	*n* = 74	*n* = 31
	17 (0.1–125)	10 (0.8–68) **(<0.01)**	21 (1.8–55) (0.78)	21 (0.1–125) **(<0.01)**	16 (0.3–47) (0.56)
Leukemic transformation *n* (%)	27 (15)	9 (18) (0.46)	1 (4) (0.1)	13 (16) (0.57)	4 (13) (0.75)
Allogeneic transplant, *n* (%)	22 (12)	6 (12) (1.0)	4 (17) (0.42)	7 (9) (0.25)	5 (16) (0.46)

Bold values indicates statistical significant *P* values (*P* < 0.05).

*post-ET MF* post-essential thrombocythmia myelofibrosis, *post-PV* Post polycythemia myelofibrosis, *PMF* primary myelofibrosis. *ESA* erythropoiesis stimulating agents, *DIPSS* *plus* dynamic international prognostic scoring system.

### Response

At a median follow up of 3.7 years (0.1–14.4 years), 178 (97%) patients have discontinued therapy. 3 and 5-year discontinuation rates were 77 and 92%, respectively with median treatment duration of 17 months for all patients. Notably, patients receiving momelotinib remained on therapy longer than patients on ruxolitinib (21 vs 10 months; *p* < 0.01). Reasons for drug discontinuation included suboptimal response or progressive disease (61%), toxicity (19%), study discontinuation by sponsor (9%), leukemic transformation (4%), death (3%) or secondary malignancy (2%). 108 (59%) of patients received subsequent therapy following discontinuation of study drug, which included JAKi therapy in 61(33%), hydrea in 16 (9%), imetelstat clinical trial in 11 (6%), thalidomide/lenalidomide/pomalidomide in 8 (4%), cladribine in 4 (2%), hypomethylating agent in 4 (2%), alisertib clinical trial in 3 (2%) and SL-401 clinical trial in 1 (1%). Overall, treatment-related grade 3 or 4 hematologic and non-hematologic adverse events were reported in 88 (48%) and 34 (19%) of patients, respectively.

Spleen response was achieved in 83 of 166 (50%) evaluable patients with median response duration of 22 months (2–132 months) and was more likely with fedratinib (83% *vs* 47%, in those receiving *vs* not receiving fedratinib, *p* < 0.01), and in the absence of *ASXL1/SRSF2* mutations (58% vs 40%, in absence *vs* presence of *ASXL1/SRSF2*, *p* = 0.04). Multivariable analysis confirmed superior spleen response with fedratinib and absence of *ASXL1/SRSF2* mutations (Table 2). Transfusion independence was achieved in 27 of 66 (47%) transfusion-dependent patients for a median of 14 months (6–82 months) and was more likely with momelotinib (51% vs 28% in those receiving *vs* not receiving momelotinib, *p* = 0.02) and in the absence of thrombocytopenia < 100 × 10^9^/l (50% vs 0%, in absence *vs* presence of thrombocytopenia, *p* < 0.01) (Table 2). Neither spleen nor anemia response was influenced by driver mutation status, unfavorable karyotype, leukocyte count >25 × 10^9^/l, peripheral blasts ≥1% and hemoglobin <10 g/dl (*p* > 0.1). In addition, symptom response was documented in 83 of 136 (60%) of patients with constitutional symptoms at baseline, response rates were 48, 57, 65, and 85% in patients receiving momelotinib, ruxolitinib, fedratinib, and BMS-911543, respectively.

**Table 2:** Predictors of inferior response and survival in 183 JAK2 inhibitor-naive patients with myelofibrosis receiving *JAK2* inhibitors in the context of clinical trials.

Variables	Spleen response	Anemia response in transfusion-dependent patients	Overall survival	Leukemia-free survival
	*N* evaluable = 166	*N* evaluable = 66	*N* evaluable = 183	*N* evaluable = 183
	Univariate/Multivariate *P-value Odds ratio*	Univariate/Multivariate *P-*value Odds ratio	Univariate/Multivariate *P-*value HR (95% CI)	Univariate/Multivariate *P-*value HR (95% CI)
Age > 65 years	0.62	0.64	<**0.01/<0.01** 3.5(2.2–5.7)	0.21
Male gender	0.11/0.72	0.37	0.42	0.79
PMF vs Post-ET/PV MF	0.34	1.0	0.65	0.92
Hemoglobin < 10 g/dl.	0.28	0.62	0.15	0.49
Leukocyte count > 25 × 10^9^/l	0.40	0.65	0.11	0.74
Peripheral blasts ≥ 1%	0.18	0.13	0.61	0.97
Platelet count < 100 × 10^9^/l	0.29	<**0.01/<0.01**	0.94	0.27
Transfusion-dependence	1.0		**<0.01/<0.01** 2.1 (1.3–3.3)	0.39
Favorable vs unfavorable karyotype	0.17	0.40	**0.02/**0.26	0.89
Absence of type 1/like *CALR* mutation	0.13/0.18	0.19	**<0.01/<0.01** 2.8 (1.3–5.8)	0.15
Presence of *ASXL1/SRSF2* mutations	0.06**/0/04** OR 0.43	0.49	**0.01/0.04** 1.6 (1.01–2.5)	0.38
Type of JAK2 inhibitor	**<0.01/0.01**	0.07**/0.02**	0.31	0.33
Ruxolitinib vs fedratinib	<**0.01/0.01** OR 0.08			
Ruxolitinib vs momelotinib	0.10**/0.04** OR 0.34			
Ruxolitinib vs BMS JAK inhibitor	**0.02/0.03** OR 0.19			
Fedratinib vs momelotinib	**<0.01/**0.07 OR 5.3	**0.04/0.02** OR 0.07		

Bold values indicates statistical significant *P* values (*P* < 0.05).

*PMF* primary myelofibrosis, *post-ET MF* post-essential thrombocythemia myelofibrosis, *post-PV* Post polycythemia myelofibrosis.

### Survival

**Fig. 1:**
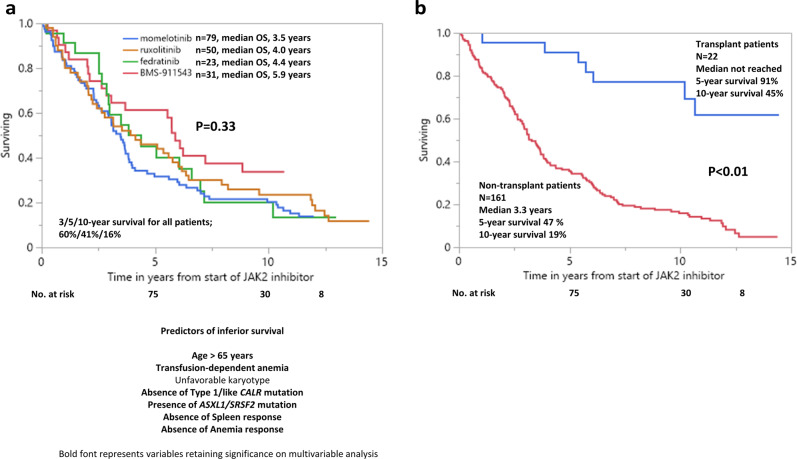
Post-treatment survival of 183 JAK2 inhibitor-naive patients with myelofibrosis receiving JAK2 inhibitors in the context of clinical trial. **a** Post-treatment survival of 183 JAK2 inhibitor-naive patients with myelofibrosis receiving *JAK2* inhibitors in the context of clinical trials, stratified by specific drug, **b** Post-treatment survival of 183 inhibitor-naive patients with myelofibrosis receiving *JAK2* inhibitors in the context of clinical trials, stratified by allogeneic transplantation.

**Fig. 2:**
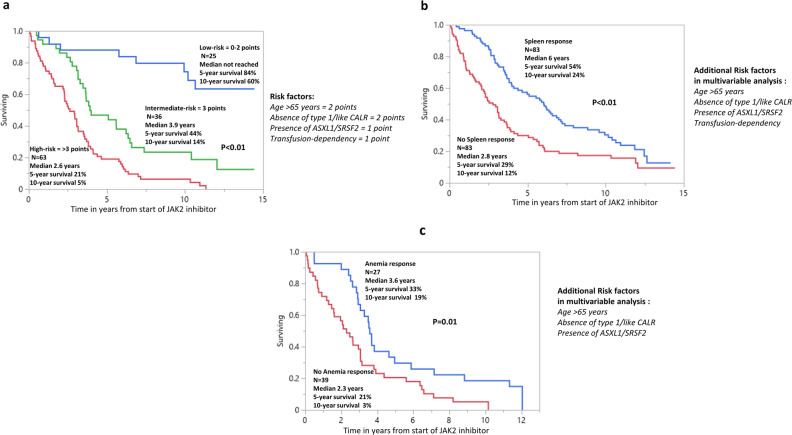
Post-treatment survival of JAK2 inhibitor-naive patients with myelofibrosis receiving JAK2 inhibitors in the context of clinical trials and informative for high-risk mutations. **a** Post-treatment survival of 124 JAK2 inhibitor-naive patients with myelofibrosis receiving *JAK2* inhibitors in the context of clinical trials and informative for high-risk mutations, stratified by HR-weighted scoring system. **b** Post-treatment survival of 166 JAK2 inhibitor-naive patients with myelofibrosis receiving *JAK2* inhibitors in the context of clinical trials and evaluable for spleen response, stratified by spleen response. **c** Post-treatment survival of 66 JAK2 inhibitor-naive patients with myelofibrosis receiving *JAK2* inhibitors in the context of clinical trials and transfusion-dependent anemia, stratified by anemia response.

All study patients were followed to death or 2022, during which time 149 (81%) deaths, 27 (15%) leukemic transformations, and 22 (12%) allogeneic stem cell transplants (ASCT) were recorded. Median overall survival was 3.7 years with 3/5/10-year survival rates of 60, 41 and 16%, respectively. Survival was similar with momelotinib, ruxolitinib, fedratinib and BMS-911543 (3.5, 4, 4.4, and 5.9 years, respectively, *p* = 0.33) (Fig. 1a.) but was prolonged in patients that underwent ASCT *vs* those not transplanted (not reached *vs* 3.3 years; *p* < 0.001, 5/10-year survival, 91%45% vs 47%19%) (Fig. 1b). In univariate analysis of pre-treatment variables, age > 65 years (*p* < 0.01), absence of type 1/like *CALR* mutation (*p* < 0.01), baseline transfusion need (*p* < 0.01), unfavorable karyotype (*p* = 0.02), and presence of *ASXL/SRSF2* mutations (*p* = 0.01) predicted inferior survival (Table 2). Multivariable analysis confirmed age >65 years (*p* < 0.01, HR 3.5), absence of type 1/like *CALR* mutation (*p* < 0.01, HR 2.8), baseline transfusion need (*p* < 0.01, HR 2.1), and presence of *ASXL1*/*SRSF2* mutations (*p* = 0.04, HR 1.6) as risk factors for inferior survival (Table 2). A subsequent HR-based survival prediction model was generated by allocation of two adverse points for age > 65 years and absence of type 1/like *CALR* mutation and one adverse point each for transfusion dependence and presence of *ASXL1/SRSF2* mutations which segregated three risk categories; low risk (0–2 points), intermediate risk (3 points) and high risk (>3 points) with 5/10-year survival rates of 84%/60%, 44%/14%, and 21%/5%, respectively (*p* < 0.01) (Fig. 2a). In addition, both spleen (6 vs 2.8 years in responders *vs* non-responders; *p* < 0.01, 5/10-year survival, 54%24% vs 29%12%) and anemia response (3.6 vs 2.3 years in responders *vs* non-responders; *p* = 0.01, 5/10-year survival, 33%19% vs 21%3%) were independently associated with improved short-term survival (Fig. 2b, c). On the other hand, in univariate analysis, leukemia-free survival was not influenced by driver mutation status, *ASXL1/SRSF2* mutations, unfavorable karyotype, leukocyte count >25 × 10^9^/l, peripheral blasts ≥1%, hemoglobin <10 g/dl, or thrombocytopenia <100 × 10^9^/l (*p* > 0.1) (Table 2). Moreover, leukemic transformation rates were similar in patients receiving momelotinib (16%), ruxolitinib (18%), fedratinib (4%), and BMS-911543 (13%) (*p* = 0.33).

## Discussion

We pioneered early clinical development of momelotinib, ruxolitinib, fedratinib and BMS-911543 JAKi for the treatment of MF, therefore the current study is uniquely poised to provide comparative data on long-term outcomes for patients treated with each JAKi [2, 5, 6, 9, 10]. The vast majority of patients were followed until death, and follow-up for living patients exceeded 14 years. The findings from the current study confirm the palliative role of JAKi therapy through reduction of splenomegaly, achievement of transfusion-independence and relief of constitutional symptoms [16]. In that regard, a novel retrospective comparison of anemia and spleen response across all four JAKi is unveiled; fedratinib was associated with higher spleen response, while anemia response was superior with momelotinib. In addition, spleen response was found to be superior in the absence of *ASXL1*/*SRSF2* mutations. Previous studies on the impact of mutations on spleen response in JAKi treated patients with MF have yielded conflicting results. In an analysis of MF patients treated with ruxolitinib in the COMFORT-2 study, spleen response was not influenced by mutational profile [17], while another study showed superior spleen response in the absence of *ASXL1/EZH2/IDH1/2* mutations in ruxolitinib treated patients [18]. On the other hand, we have previously reported superior spleen and anemia response in the absence of *ASXL1* mutations in momelotinib treated patients with MF [19, 20].

Additional noteworthy observations include high treatment discontinuation rates, with less than 5% of study patients remaining on long-term therapy (>10 years). Importantly, two-thirds of patients discontinued therapy due to suboptimal response or disease progression, while death or leukemic transformation on-study was documented in a minority (<5%) of patients. In our series, the high discontinuation rates secondary to toxicity was likely a result of exposure to higher drug dosage in the phase-1 studies. The high treatment discontinuation rates attest to the transient benefit from JAKi therapy and although short-term survival was superior in spleen and anemia responders, long-term survival was not impacted by neither achievement of spleen nor anemia response with 10-year survival rates of <25%. Moreover, we have recently shown that the short-term survival benefit associated with anemia response in momelotinib treated MF patients might be limited to those with unfavorable genetic profile [21]. It is to be noted that patients remained on momelotinib longer and had higher anemia response rates compared to ruxolitinib, despite which survival was similar, which is likely due to momelotinib treated patients being older and belonging to higher DIPSS-plus risk.

The subject of whether MF patients derive long-term survival benefit from JAKi therapy remains contentious. A pooled analysis of the COMFORT-1 and 2 studies and a recently published prospective real-world series suggested prolonged survival in ruxolitinib-treated patients [22, 23]. In contrast, patients treated on momelotinib and ruxolitinib clinical trials at the Mayo Clinic did not show a significant survival advantage when compared to risk-adjusted patient cohorts not receiving JAKi therapy [19, 24]. The current study reinforces the prognostic impact of previously established clinical and molecular risk factors for survival in MF patients treated with JAKi, namely age >65 years, absence of type 1/like *CALR* mutation, presence of *ASXL1/SRSF2* mutations, and transfusion dependence [15, 25]. Based on the aforementioned variables, we propose a practical three-tiered survival prediction model for JAKi-treated MF patients, which enables identification of long-lived patients with 10 year survival of 60%. On the other hand, MF patients categorized as “high-risk” had considerably shortened long-term survival with 10-year survival of 5%, despite receiving JAKi therapy. Although achievement of anemia and spleen response improved short-term survival, a durable survival benefit was secured only by ASCT, which could have possibly resulted from ASCT candidacy itself by favoring younger patients with fewer medical comorbidities. Limitations of the current study include the retrospective design, which resulted in our inability to account for the impact of subsequent therapies on survival. Taken together, our findings underscore the transient benefit derived from JAKi therapy in MF in terms of palliation of splenomegaly and constitutional symptoms, however, impact on long-term survival was limited and primarily determined by pre-treatment clinical and molecular risk profile. Accordingly, early ASCT referral for patients with higher risk MF is advised.

## Supplementary information

checklist

## Data Availability

Please email the corresponding author.
